# Ex vivo magnetic particle imaging of vascular inflammation in abdominal aortic aneurysm in a murine model

**DOI:** 10.1038/s41598-020-69299-y

**Published:** 2020-07-24

**Authors:** Dilyana B. Mangarova, Julia Brangsch, Azadeh Mohtashamdolatshahi, Olaf Kosch, Hendrik Paysen, Frank Wiekhorst, Robert Klopfleisch, Rebecca Buchholz, Uwe Karst, Matthias Taupitz, Jörg Schnorr, Bernd Hamm, Marcus R. Makowski

**Affiliations:** 1 Department of Radiology, Charité-Universitätsmedizin Berlin, Corporate Member of Freie Universität Berlin, Humboldt-Universität zu Berlin and Berlin Institute of Health, Charitéplatz 1, 10117 Berlin, Germany; 2 Department of Veterinary Medicine, Institute of Veterinary Pathology, Freie Universität Berlin, Robert-von-Ostertag-Str. 15, Building 12, 14163 Berlin, Germany; 3 Department of Veterinary Medicine, Institute of Animal Welfare, Animal Behavior and Laboratory Animal Science, Freie Universität Berlin, Königsweg 67, Building 21, 14163 Berlin, Germany; 4 Department 8.2-Biosignals, Physikalisch-Technische Bundesanstalt Berlin, Abbestrasse 2-12, 10587 Berlin, Germany; 5 Department of Diagnostic and Interventional Radiology, Technische Universität München, Ismaninger Str. 22, 81675 Munich, Germany; 6 Institute of Inorganic and Analytical Chemistry, Westfälische Wilhelms-Universität Münster, Corrensstr. 30, 48149 Münster, Germany

**Keywords:** Diagnostic markers, Inflammation, Medical imaging, Medical research, Molecular medicine

## Abstract

Abdominal aortic aneurysms (AAAs) are currently one of the leading causes of death in developed countries. Inflammation is crucial in the disease progression, having a substantial impact on various determinants in AAAs development. Magnetic particle imaging (MPI) is an innovative imaging modality, enabling the highly sensitive detection of magnetic nanoparticles (MNPs), suitable as surrogate marker for molecular targeting of vascular inflammation. For this study, Apolipoprotein E-deficient-mice underwent surgical implantation of osmotic minipumps with constant Angiotensin II infusion. After 3 and 4 weeks respectively, in-vivo-magnetic resonance imaging (MRI), ex-vivo-MPI and ex-vivo-magnetic particle spectroscopy (MPS) were performed. The results were validated by histological analysis, immunohistology and laser ablation-inductively coupled plasma-mass spectrometry. MR-angiography enabled the visualization of aneurysmal development and dilatation in the experimental group. A close correlation (R = 0.87) with histological area assessment was measured. Ex-vivo-MPS revealed abundant iron deposits in AAA samples and ex-vivo histopathology measurements were in good agreement (R = 0.76). Ex-vivo-MPI and MPS results correlated greatly (R = 0.99). CD68-immunohistology stain and Perls’-Prussian-Blue-stain confirmed the colocalization of macrophages and MNPs. This study demonstrates the feasibility of ex-vivo-MPI for detecting inflammation in AAA. The quantitative ability for mapping MNPs establishes MPI as a promising tool for monitoring inflammatory progression in AAA in an experimental setting.

## Introduction

Cardiovascular diseases are currently one of the leading causes of death in the Western world. An abdominal aortic aneurysm (AAA) is defined as a weakening and dilatation of the abdominal aorta, prevailing in the infrarenal portion of the artery. The prevalence of AAA has been on the rise during the last decades as a result of demographic ageing, screening programs and improved clinical imaging techniques^1^. The main risk of undetected, asymptomatic aneurysms is progressive expansion, followed by rupture, hemorrhage and death in approximately 80% of the cases^2^. The development of AAA involves inflammation as a fundamental process. Chronic inflammation is characterized by the infiltration of inflammatory cell types, mainly macrophages and monocytes in the thrombus and throughout all layers of the aortic wall^3^. These cells release several proteolytic enzymes such as matrix metalloproteinases, cytokines and oxidation-derived free radicals, leading to vascular smooth muscle cell apoptosis and degradation of the aortic tunica media^3^. In AAA, elastolysis as a result of the chronic inflammation leads to reduced stability and consequently gradual dilatation of the aorta^4^. An increase in the inflammatory response and extracellular matrix (ECM) degradation results in an increased risk of rupture^5^.

Magnetic nanoparticles (MNPs) represent a molecular imaging probe type, mainly used for magnetic resonance imaging (MRI). MNPs are comprised of small iron oxide crystals, typically surface-modified by coating (polysaccharides, polyethylene glycol) or capping (organic acids). Their sensitivity to external magnetic fields and small size, ranging from 20 to 150 nm makes them ideal candidates for tracking tumor cells, drug delivery and detecting endothelial inflammation^6,7^. MNPs are internalized into macrophages/monocytes and represent a promising target for molecular imaging of inflammation and predicting aneurysm growth^8–10^. Ferucarbotran (RESOVIST) is a clinically approved MNP for MRI, designed for detecting liver lesions. It consists of magnetic nanoparticles (Magnetite–Fe_3_O_4_/Maghemite–Fe_2_O_3_) coated with carboxydextran. Previous studies have proven ferucarbotrans efficiency for imaging AAA in clinical studies^11^ as well as in animal models^12^. No adverse reactions are associated with rapid intravenous (i.v) injection of ferucarbotran^13^.

So far, there are several different approaches for detecting inflammation in AAA. 18F-fluorodeoxyglucose (FDG) is a radiopharmaceutical tracer for positron emission tomography (PET) and PET computed tomography (CT), targeting high-glucose-using cells including macrophages in aneurysm inflammatory sites, yet it relies on ionizing radiation^14^. In recent years, molecular MRI targeting macrophages and monocytes via various types of MNPs has gained momentum^9^. However, the detection of MNPs in MRI is highly dependent on the surrounding tissue and can be hard to distinguish from other sources (e.g. air, imaging artifacts or pathological tissue changes). Additionally, a reference scan before MNP injection is required to perform quantification.

Magnetic particle imaging (MPI) is a novel tomographic imaging modality for the highly sensitive detection and quantification of magnetic nanoparticles^15^. First described by Gleich and Weizenecker in 2005 MPI is currently used in preclinical studies and is not yet in clinical practice. Opposed to MRI, where MNPs cause signal void, MPI detects MNP tracers directly, causing positive contrast without any background signal from the surrounding tissue. MPI images are comparable to the images known from nuclear medicine imaging modalities such as PET and single-photon emission computed tomography (SPECT). Compared to MRI, MPI has a much higher sensitivity in detection of MNPs. MPI enables imaging and quantification of MNPs with higher specificity and without the need of additional measurements before injection of MNPs. MPI scanners have a spatial resolution in the millimeter range, which compares well with the resolution of clinical PET and SPECT. In contrast to PET or SPECT, MPI provides a much higher temporal resolution by using non-radiating tracers. Therefore, MPI makes a promising candidate for vascular imaging.

In this study, we assessed the potential of sensitive ex vivo MP imaging for the characterization of relevant parameters in AAA development and progression. A MPI-suitable MNP, ferucarbotran was used to evaluate the inflammatory processes in the aortic wall.

## Results

**Figure 1:**
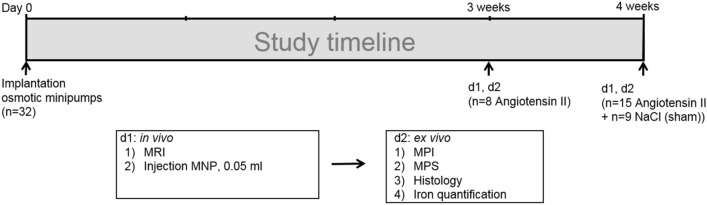
Experimental setup. Native in vivo MR imaging (N = 32) was performed after three (group 1, n = 8) and four weeks (group 2, n = 15) of angiotensin II infusion. Following the scan, 50 µl dose of macrophage-specific iron-oxide particles (ferucarbotran, 46.66 µg iron per kg body weight) was administered via the tail vein. Ex vivo analysis (magnetic particle spectroscopy, magnetic particle imaging, histology, immunohistochemistry, laser ablation coupled to inductively coupled plasma-mass spectrometry) was performed 24 h after MNP administration. The control group consisted of apolipoprotein E-deficient mice (n = 9) implanted with osmotic minipumps filled with sodium chloride, serving as the control group. *MPI* magnetic particle imaging, *MPS* magnetic particle spectroscopy, *MNP* magnetic nanoparticles.

**Figure 2:**
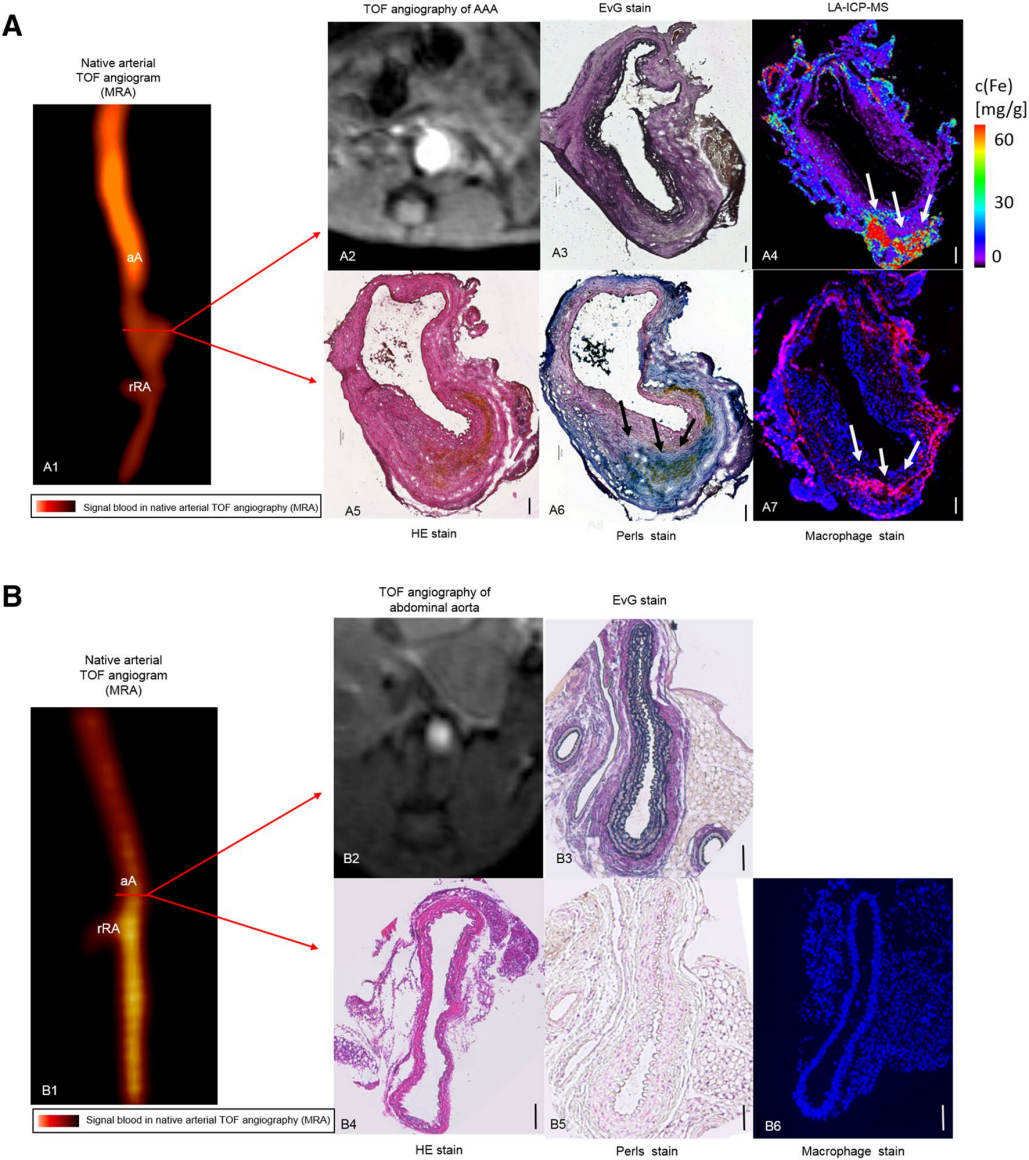
In vivo MRI of inflammatory activity during the development of aortic abdominal aneurysm compared to an animal from the control group. **(A1)** Time-of-flight angiogram showing the suprarenal abdominal aorta, including the right renal artery, of a male apolipoprotein E-deficient (Apo E −/−) mouse after four weeks of angiotensin II (Ang II) infusion. (**A2)** A pronounced dilatation of the arotic lumen was observed on the T1 weighted sequence after 4 weeks of angiotensin infusion. (**A3–A7)** Ex vivo histological measurements using EvG (**A3**), LA-ICP-MS (**A4**), HE (**A5**), Perls stain (**A6**) confirmed the in vivo findings. (**A4, A6, A7)** A strong correlation between the areas positive for iron-oxide particles in LA-ICP-MS (**A4**), Perls’ stain (**A6**) and immunofluorescence for macrophage accumulation (**A7**) in corresponding histological sections was observed. The scale bars represent 100 μm. (**B1)** Time-of-flight angiogram showing the suprarenal abdominal aorta, including the right renal artery, of a male apolipoprotein E-deficient (Apo E −/−) control group mouse after four weeks of sodium chloride solution infusion. (**B2)** No dilatation of the aortic lumen was observed on the T1 weighted sequence after 4 weeks of sodium chloride solution infusion. (**B3–B6)** Ex vivo histological measurements using EvG (**B3**), HE (**B4**), Perls’ Prussian Blue (**B5**) and immunofluorescence for macrophage accumulation **(B6)** in corresponding histological sections revealed neither MNP accumulation nor AAA development. The scale bars represent 100 μm. *TOF* arterial time of flight, *aA* suprarenal abdominal aorta, *rRA* right renal artery, *MRA* magnetic resonance angiography, *HE* hematoxylin–eosin-staining, *EvG* Miller’s elastica van Gieson staining, *LA-ICP-MS* laser ablation coupled to inductively coupled plasma-mass spectrometry, *MNP* magnetic nanoparticles.

No side effects or adverse reactions to the imaging agents were observed in the investigated animals. In the control group, consisting of sham-operated mice (n = 9) that received a continuous saline infusion for 28 days, AAA development was not observed. In the experimental group (n = 23), the continuous infusion of angiotensin II (Ang II) via osmotic minipumps led to the formation of suprarenal aortic aneurysms (Figs. 1, 2). Animals developing no abdominal aneurysms were excluded from the study prior to data acquisition (n = 4).

### MR angiography of abdominal aortic aneurysms

**Figure 3:**
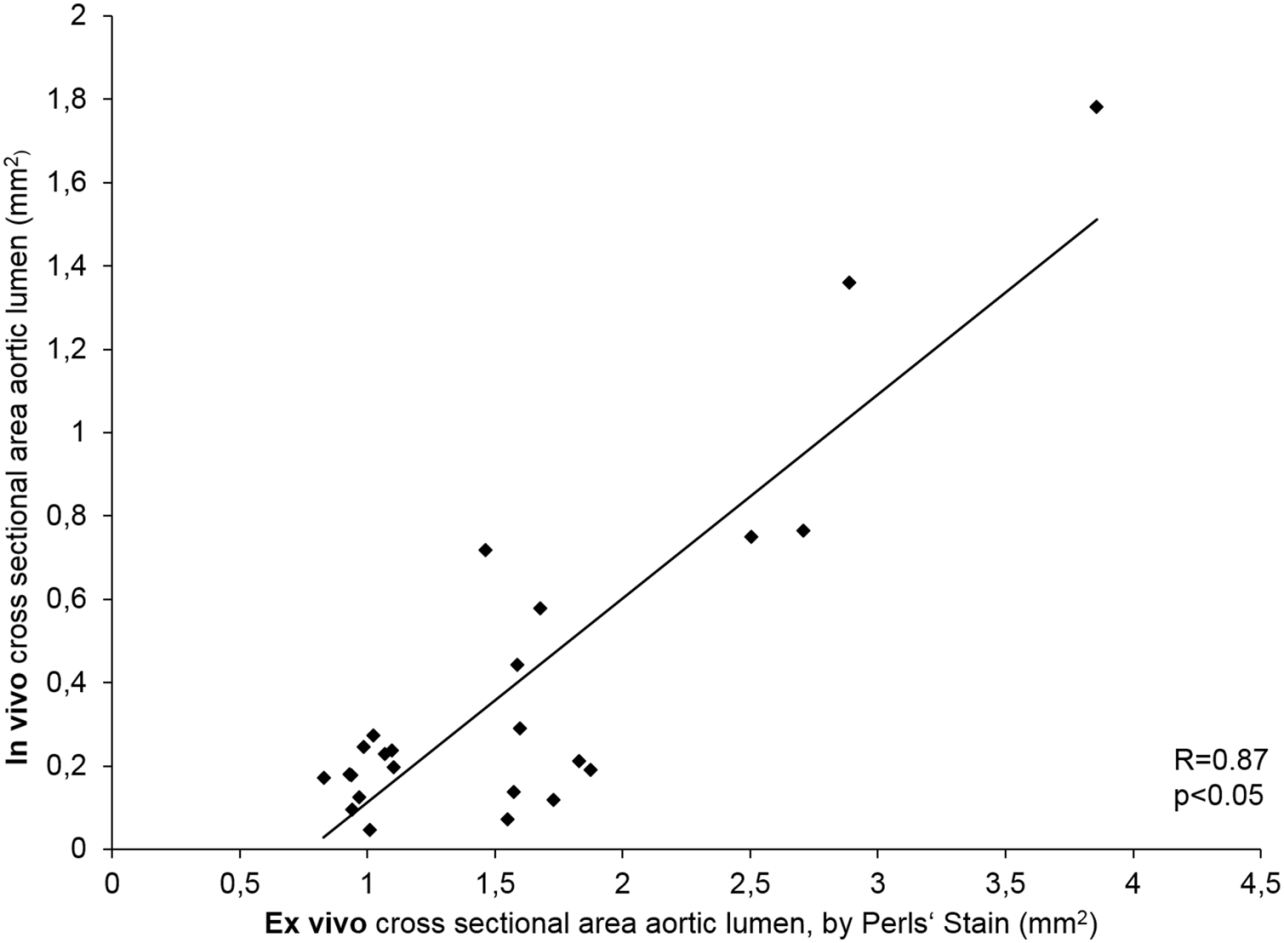
Correlation of in vivo MRI and ex vivo histological cross sectional AAA area measurements. To investigate the presence of AAA, in vivo MRI findings were compared to histological cross sections from the same region of the aorta. Time-of-flight angiogram detected the development of AAA in the experimental group. A strong correlation (R = 0.87) between the in vivo MRA and ex vivo histology images was shown. Overall, these measurements indicate an excellent agreement between in vivo and ex vivo measurements of the lumen dilatation in AAA. *MRA* magnetic resonance angiography.

**Figure 4:**
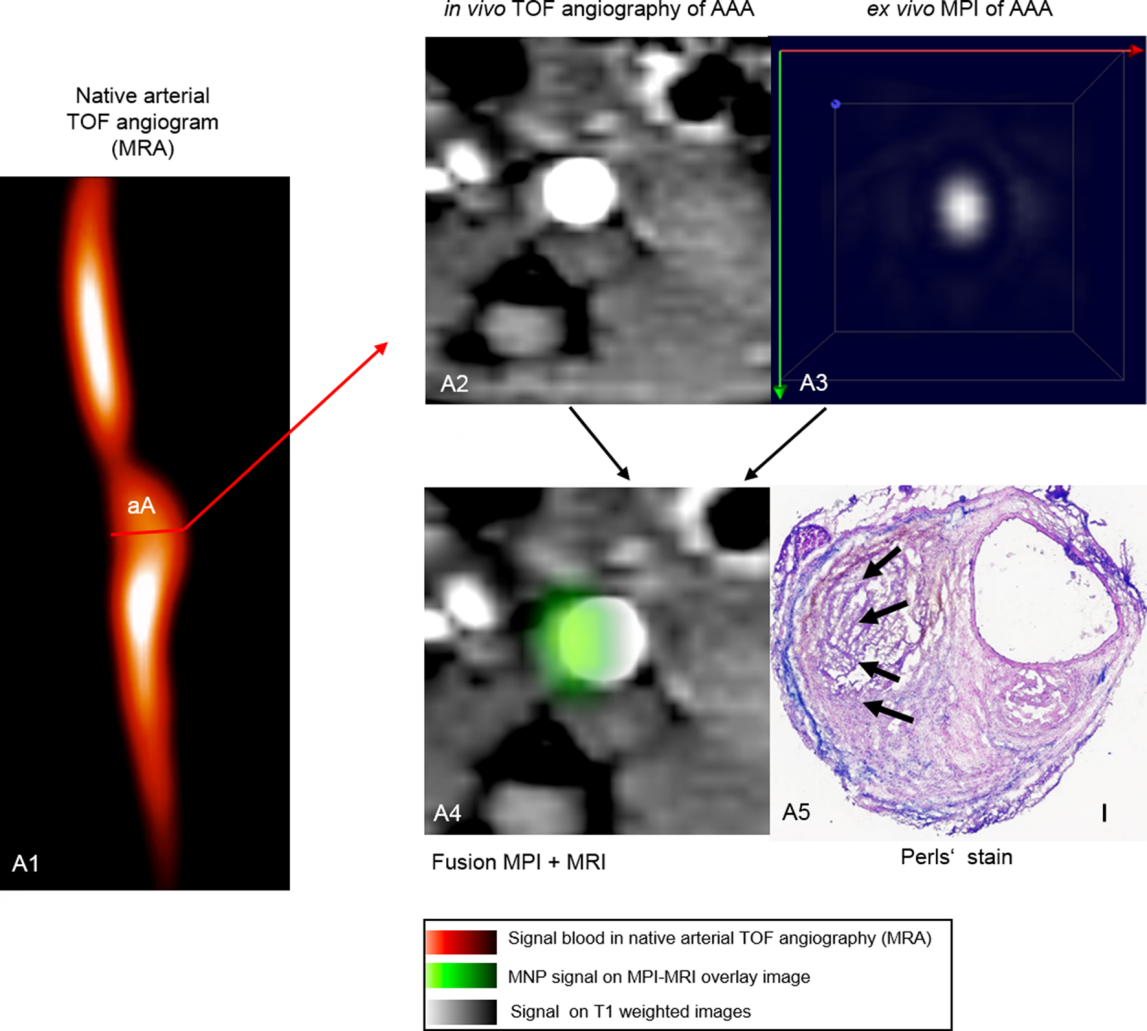
In vivo MRI and ex vivo MPI of inflammatory-activity during the development of aortic abdominal aneurysm. (**A1)** Time-of-flight angiogram showing the suprarenal abdominal aorta of a male apolipoprotein E-deficient (Apo E −/−) mouse after four weeks of angiotensin II (Ang II) infusion; (**A2)**: a pronounced dilatation of the arotic lumen was observed on the T1 weighted sequence after 4 weeks of angiotensin infusion; (**A3)** ex vivo MPI of the AAA region of the same mouse; (**A4)**: ex vivo aortic MPI—in vivo whole body MRI signal manual fusion overlay based on anatomical landmarks; (**A5)** Perls’ Prussian Blue; the scale bar represents 100 μm. *TOF* arterial time of flight. *aA* suprarenal abdominal aorta, *MRA* magnetic resonance angiography, *MNP* magnetic nanoparticles.

Cross-sections of the abdominal aorta were assessed after 3 and 4 weeks of Ang II infusion (Fig. 1). The protocol included scans prior to administration of ferucarbotran. A significant aortic diameter increase was visible in T1 3D TOF (p < 0.05) (Figs. 3, 4), while no difference was seen in the control animal group. The aortic diameter increased by 88% percent in the 3-week group and 175% in the 4-week group.

### Ex vivo magnetic particle imaging of AAA

**Figure 5:**
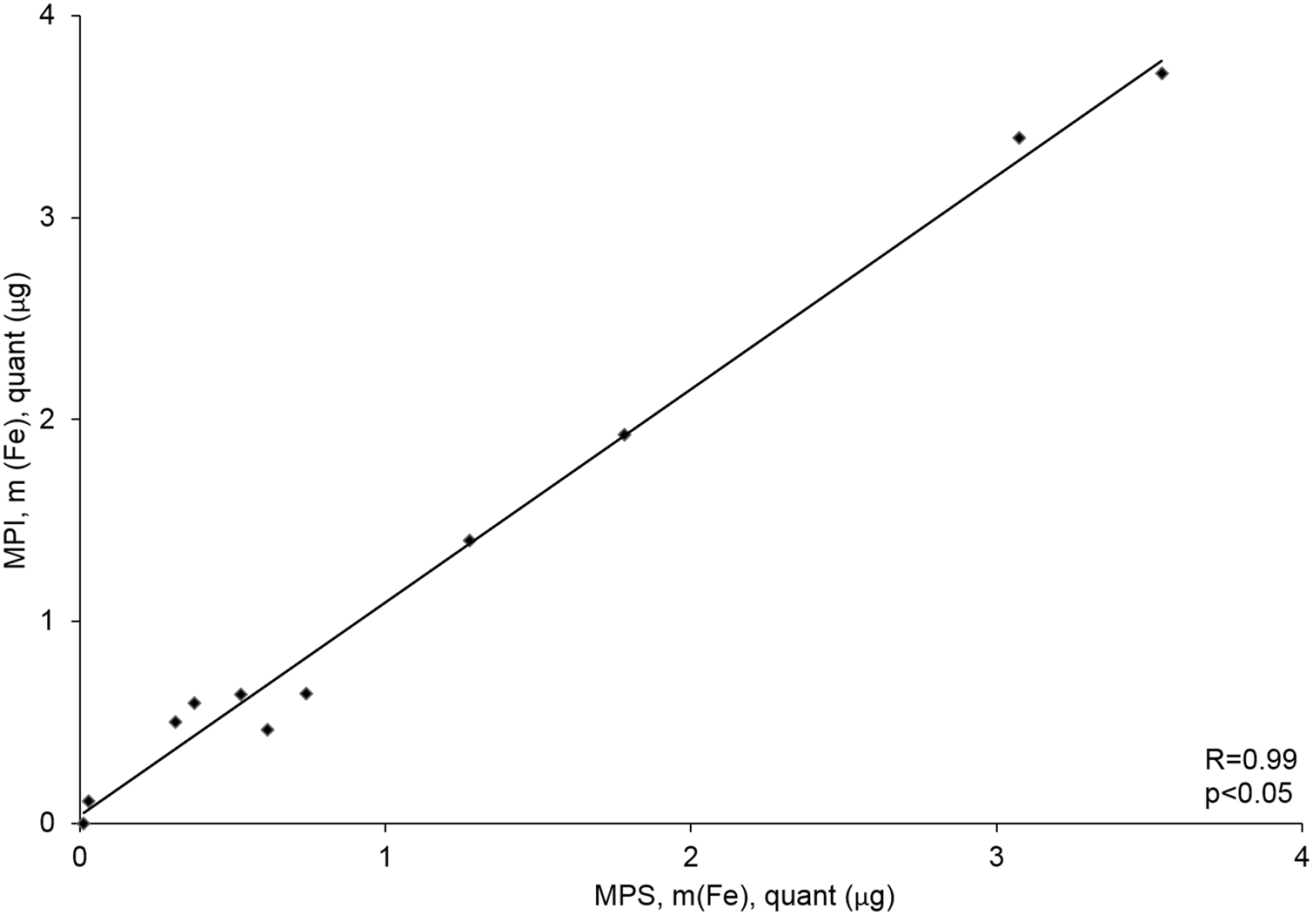
Correlation of ex vivo MPS and MPI iron oxide particle measurements. MPS is a standard method for validation of MPI results, combining static and dynamic magnetic properties of iron oxide nanoparticles. A strong correlation (R = 0.99) between the amount of iron-oxide particles measured in ex vivo MPS and ex vivo MPI was measured. *MPS* magnetic particle spectroscopy, *MPI* magnetic particle imaging.

To evaluate the potential of MPI for measuring inflammatory response in AAA, ex vivo MPI images of the aorta were acquired 24 h after i.v. ferucarbotran administration. The AAAs with an overall iron content above 0.3 µg were visible in MPI (Fig. 4A3). The reconstruction of images of ex vivo AAAs with low iron content was possible only with the immobilized MNP system function (SF). The decay of higher harmonics in freeze-dried ferucarbotran is rapid in comparison to the fluid sample. Hence, fewer frequency components were used in the reconstruction, which is not beneficial in sense of MPI resolution. However, the immobilized state of MNPs in the AAA after phagocytosis by macrophages ^16^ resembles more the immobilized state of MNPs in sugar matrix of mannitol SF and results in a more reliable reconstruction. The magnetic particle spectroscopy (MPS) quantification results are in good agreement and validate the MPI iron mass quantification (R = 0.99) (Fig. 5). The average deviation of total iron amount determined by MPI from MPS is 8.3% for samples above 1 µg and 20.6% for samples below 1 µg and 15.2% overall. The slight over– or underestimation of iron amount in comparison to MPS results might arise from partial volume effects due to the limited resolution of MPI and low signal-to-noise ratio (SNR).

### Histological analysis

Elastica van Gieson (EvG) stained histological sections revealed strong extracellular matrix remodeling, visible in the 3 weeks- as well as in the 4 weeks Ang II infusion groups (Fig. 2A3). Degradation of elastic fibers and following dilatation of the aortic lumen was accompanied by the formation of a thrombus. On the other hand, elastogenesis characterized by a higher amount of elastic fibers in the areas adjacent to the vascular lumen was observed, indicating a repair process in late stage aneurysm. The Perls’ Prussian blue stained histopathologic sections revealed abundant iron within AAA, while no or little iron was detected in the control group (Fig. 2A6). Anti-CD68 monoclonal antibody immunohistology analysis revealed abundant macrophage accumulation in the adventitial area of the aneurysm (Fig. 2A7).

### Correlation of magnetic particle spectroscopy, magnetic particle imaging, histology and immunohistochemistry

MPS functions as a zero-dimensional MPI with higher sensitivity. The accumulation of iron in the AAAs was measured with MPS and results were used for validation of the MPI quantification.

Ex vivo MPI and MPS measurements revealed abundant iron within AAAs (Figs. 4, 5), while no or little iron was detected in the control group (Fig. 2B5).

**Figure 6:**
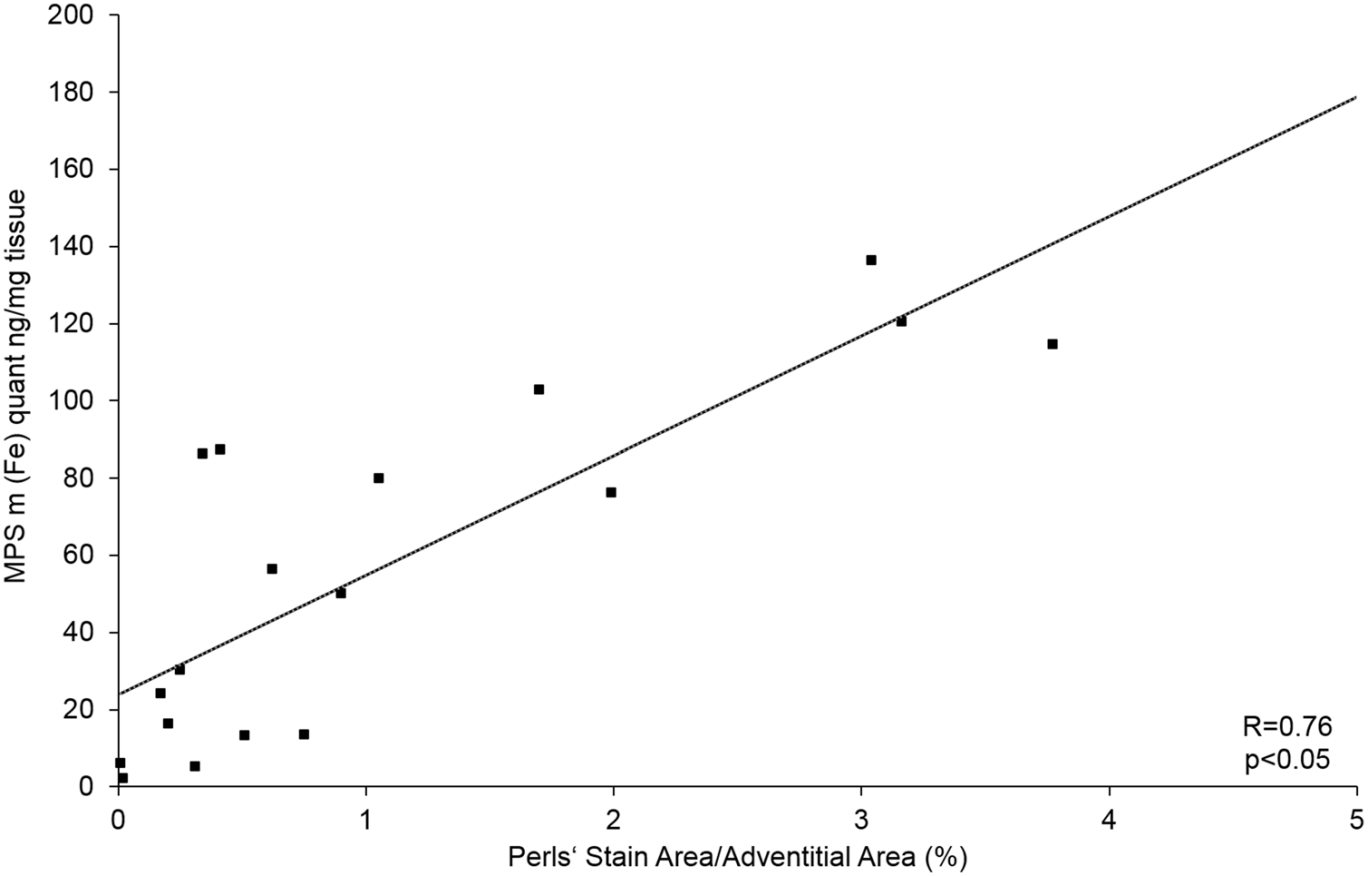
Correlation of MPS iron oxide particle measurements and Perls’ Prussian Blue stained histological measurements. In order to investigate the absolute amount of iron oxide particles in AAA samples, ex vivo MPS measurements were performed prior to histological processing. A strong correlation between the amount of iron-oxide particles measured in ex vivo MPS and Perl’s Prussian Blue staining was shown. The combined assessment of both analytical methods verify the feasibility of MPS for detection of inflammation sites in AAA. *MPS* magnetic particle spectroscopy.

A strong correlation was found between the areas positive for CD68 immunohistology stain and Perls’ Prussian blue stain (Figs. 2A6, A7), confirming the co-localization of macrophages and MNPs. There is a positive correlation between the amount of iron measured in Perls’ Prussian Blue and ex vivo MPS as well as ex vivo MPI (Figs. 5, 6) iron quantification. The MPS quantification of the intact harvested AAA verified that the stained iron in the sections does not originate from endogenous iron nor from cutting blades during tissue processing.

### Elemental bioimaging by means of laser ablation-inductively coupled plasma-mass spectrometry for spatial localization of iron

To determine the spatial distribution of MNPs within the aneurysmal wall, laser ablation inductively coupled plasma mass spectrometry (LA-ICP-MS) measurements were performed in two mice after 4 weeks of Ang II infusion. A pronounced colocalization of iron, macrophages (CD68) and Perls’ Prussian blue positive areas was found (Fig. 2A4, A6, A7).

## Discussion

This study demonstrates the feasibility of ferucarbotran-enhanced ex vivo MPI for the detection of vascular inflammation in AAA. Ex vivo iron measurements via LA-ICP-MS, immunohistology, histopathology, MPI and MPS demonstrated a strong correlation, thus confirming the inflammatory activity and resulting MNP accumulation in the aneurysmal wall.

Inflammation is a key process in the emergence and development of many pathological conditions such as vascular disease, cancer, immune and neurologic disorders. Tracking inflammatory processes usually involves low specificity biomedical imaging or invasive methods such as biopsies^17^. Previous research on inflammation detection has tended to focus on MR imaging, demonstrating the MNP uptake at inflammation sites^18,19^. A major disadvantage of this method is that iron oxide nanoparticles cause a signal intensity decrease in MRI, which could easily be missed or mistaken for an artifact^17^. MPI allows the detection of inflammation through MNPs with a higher specificity and SNR.

Ferucarbotran is currently considered the standard MPI agent due to its commercial availability and excellent signal properties^20,21^. Uptake of MNPs in AAAs has previously been investigated^22^ and imaged with MRI in murine model as well as in human patients^10,16,23^. It has been demonstrated that MNP-enhanced MRI could identify aortic wall inflammation in patients with AAAs and predicts the rate of aneurysm growth and clinical outcome^10,22^. In a clinical study, the growth rate of AAA of patients correlated significantly with amount of MNP in the aneurysmal wall, despite comparable aneurysm diameters^10^.

Further experimental investigations should focus on enhancing the quality of angiographic and vascular MPI. MNPs are administered mostly i.v., allowing the direct visualization of the blood flow^24^. The first dynamic MPI-static MRI imaging sequence of a beating mouse heart was acquired in 2009^25^. Since most MPI scanners have no tissue depth limitation, imaging the whole cardiovascular system without signal attenuation in a similar manner is theoretically possible^24^. Regarding AAA, so far only hemodynamic aneurysm phantom experiments have been conducted, paving the way for in vivo MPI AAA imaging in the future. Once MNPs are administered i.v., there is a very limited imaging time frame before the particles escape the cardiovascular system and start accumulating in the spleen and liver^26,27^. However, for many diseases models, including AAA a prolonged blood circulation time would be beneficial. Ferumoxytol for example, an ultrasmall superparamagnetic iron oxide (USPIO) circulates longer^28^, avoiding uptake by Kupffer cells in the liver and also reducing the chances for shadowing effect in MPI^29^. The prolonged circulation is favorable for access to the AAA site and uptake by macrophages. Future studies should target identifying further, suitable, high resolution MPI tracer. Furthermore, we should also consider the application of MPI for AAA rupture prediction. In a MRI study by Brangsch et al.in a murine AAA model, the prediction of aneurysm rupture with an iron oxide based contrast agent was associated with a sensitivity of 80% and specificity of 89% . It is a question of future research to investigate whether these results are also applicable to MPI.

Although the pathophysiology of AAA is not completely deciphered, it is a well-known fact that both inflammatory activity, characterized by a pronounced proinflammatory cell infiltration, as well as ECM degradation, defined by breakdown of cross-linked elastin and collagen are key^16^. While those two mechanisms appear to be autonomous, the formation of AAA is most likely when they co-occur.

Inflammatory activity is an excellent in vivo indicator for the characterization of AAAs. Previous studies from the past years have showcased the potential of MNPs for imaging macrophage activity in AAAs^10,23,30,31^. There are two different types of macrophages, namely M1 and M2^32^. While M1 macrophages are associated with proinflammatory cytokines, classically activated by interferon γ (IFN-γ) or lipopolysaccharides (LPS) from viral and bacterial pathogens, M2 macrophages are responsible for tissue repair and wound healing, inducing collagen production^33^. AAAs are marked by a high M1:M2 cell ratio^34^, suggesting that various inflammatory cytokines secreted by M1 macrophages such as migration inhibitory factor (MIF-1) and tumor necrosis factor (TNF) in addition to matrix metalloproteinases (MMP-9) are responsible for the degradation of ECM proteins in the vessel wall, causing continuous dilatation of the aortic lumen^35,36^. In this study, we could detect and quantify the macrophage activity in AAA through several different ex vivo methods. Future work should concentrate on targeting the long-term quantification of inflammatory activity at multiple stages of the AAA development through MPI.

This study has two primary limitations. First, the strong shadowing effect^29^ observed in ferucarbotran-enhanced MPI is responsible for the absence of in vivo MPI in our experiment. When two objects with a large difference in iron concentration simultaneously present in the field of view, the object with lower iron content is suppressed and thus not visible ^37^. This is the case with the abdominal portion of the aorta in vicinity of the liver, where ferucarbotran is mainly sequestered by the reticuloendothelial system of the body.

Second, since no topological anatomical information is obtained from the MPI scans, a reference image is required from another imaging modality such as CT or MRI for accurate localization of the imaging area. Co-registration via fiducial markers^38^ is an option, however the transferring of animals from the MPI scanner to the other modality might cause spatial confidence issues and requires image post-processing. An integrated hybrid system combining MPI and MRI will assure spatial co-registration accuracy. A MPI/MRI Hybrid imaging system has been realized successfully, yet only used for static images and 2D phantom measurements^39,40^.

## Conclusion

To our knowledge, this is the first study that demonstrates the potential of a combined in vivo MR—ex vivo MP imaging for the assessment of inflammatory response in the aneurysmal wall of an Ang II-infused ApoE −/− mouse model using the MNP ferucarbotran. Future research could examine the feasibility of a combined MR-MP imaging in order to improve the in vivo characterization of AAAs.

## Methods

### Animal experiments

The animal experiments were approved and performed according to the local Guidelines and Provisions for Implementation of the Animal Welfare Act by Charite Universitaetsmedizin Berlin, the regulations of the Federation of Laboratory Animal Science Associations (FELASA) and the local animal protection committee of the LaGeSo, Berlin, Germany.

All procedures in this study were conducted by a veterinarian, and all possible steps were taken to avoid suffering at each stage of the experiment. The animals were fed with a standard lab diet and housed in a clean barrier. For surgery and for the imaging sessions, mice were anesthesized with an intraperitoneal (i.p.) combined injection of 500 µg/kg medetomidin, 50 µg/kg fentanyl, and 5 mg/kg midazolam. In order to accelerate recovery time, anesthesia was antagonized using an i.p. combination of Atipamezole (2.5 mg/kg), Naloxone (1,200 µg/kg), Flumazenil (500 µg/kg) following MRI imaging. AAAs were induced in 23 male, Apolipoprotein E deficient mice (B6.129P2-ApoE^tm1Unc^/J) mice (8 weeks old)**.** Osmotic minipumps (Alzet model 2004, Durect Corp) were implanted subcutaneously in the dorsal neck area. Angiotensin II was continuously infused with a rate of 1,000 ng/kg/min for 3 weeks (group 1, n = 8) or 4 weeks (group 2, n = 15), respectively. Sham-operated ApoE−/− mice (n = 9) delivered saline over 4 weeks serving as the control group.

In order to verify the development of AAA, native MR imaging was performed after 3 weeks (group 1) or 4 weeks (group 2 and control group), followed by i.v. injection of ferucarbotran to the tail vein (50 µl ferucarbotran, 46.66 µg iron per kg body weight). 24 h later, animals were sacrificed and the abdominal part of the aorta was harvested in order to correlate the in vivo MRI findings with ex vivo data (MPI, MPS, histology, immunohistochemistry and LA-ICP-MS).

### Magnetic nanoparticles

Ferucarbotran (RESOVIST, I’rom Pharmaceutical Co Ltd, Tokyo, Japan) is the second clinically approved MNP developed for contrast-enhanced MRI of the liver. It is a hydrophilic colloidal solution of γ-Fe_2_O_3_ coated with carboxydextran, composed of clusters of single-domain nanoparticles. Ferucarbotran has a bimodal size distribution with mean core diameters of about 4 nm and 16 nm (electron microscopy)^13,20^ and shows a mean hydrodynamic diameter (D_H_) of 60 nm (photon correlation spectroscopy)^41^. The carboxydextran coating (27–35 mg/ml with an iron to carboxydextran ratio of 1:1 (w/w)) ensures aqueous solubility of the microparticles and prevents aggregation. Ferucarbotran contains 0.5 mol Fe/l, including 40 mg/ml mannitol and 2 mg/ml of lactatic acid, adjusted to a pH of 6.5. At 37 °C, the solution has an osmolality of 0.319 osmol/kg H_2_O and a viscosity of 1,031 MPas. Upon i.v. application, ferucarbotran is taken up by the reticuloendothelial system (RES), mostly in the liver (80%) and spleen (8–9%). Following uptake in RES cells, the carboxydextran coating decomposes and the iron is conveyed to the iron pool via transferrin^42^.

## In vivo magnetic resonance imaging

Mice were imaged in supine position after induction of anesthesia. Using a clinically approved single loop coil (47 mm Siemens Healthcare Solutions, Erlangen, Germany)), the imaging sessions were performed on a clinical 3 T Siemens system (Biograph-mMR, Siemens Healthcare Solutions, Erlangen, Germany). Body temperature (37 °C) was monitored using a MR-compatible heating system (Model 1025, SA Instruments Inc, Stony Brook, NY). In conclusion to the acquisition of native scans, ferucarbotran was administered via a 30G cannula attached to a small diameter tube inserted into the tail vein of the animals.

A non-contrast-enhanced two-dimensional time-of-flight angiography (2D TOF) following a three-dimensional (3D) gradient echo scout scan was performed in transverse orientation for visualization of the abdominal aorta. Following parameters were used: field of view (FOV) of 200 × 200 mm, matrix of 960 × 960, resolution of 0.2 × 0.2x0.5 mm, 40 slices, repetition time(TR)/echo time (TE) of 35 ms/4.44 ms, flip angle of 90°, and bandwidth of 124 Hz/Px. To obtain an arterial angiogram of the abdominal aorta for planning the subsequent MR angiography, a maximum intensity projection (MIP) was automatically produced.

### Ex vivo magnetic particle imaging

Ex vivo MP imaging of the aorta was performed 24 h post-i.v. administration of 50 µl ferucarbotran after 3 (group 1, n = 8) respectively 4 (group 2, n = 15) weeks of Ang II perfusion. MPI images were acquired on a commercial preclinical MPI system (Bruker MPI 25/20 FF), equipped with a separate gradiometric receive coil ^43,44^ for improved sensitivity and image quality.

This field-free-point (FFP) based MPI system requires a pre-recorded (SF) for image reconstruction^45,46^. The SPIONs inside the FOV are excited with three drive fields at amplitudes of 12 mT, orthogonal to one another, operating at slightly different excitation frequencies (2.5 MHz divided by 102/96/99 in x-/y-/z-direction) to generate the Lissajous trajectory of the FFP movement in a selection field gradient at strength of 2.5 T/m in z-direction, and 1.25 T/m in x and y direction. The FFP scans the FOV along this Lissajous trajectory. In this matter the FOV is scanned into a 3D image of the MNP distribution at a temporal resolution of 21.5 ms.

### Magnetic particle imaging system function

The SF was measured for ferucarbotran in two forms: one in an aqueous suspension and one in a freeze-dried mannitol sugar matrix. Both SF samples, the aqueous suspension SF sample and the immobilized SF sample (in mannitol sugar matrix) ^47^ had an iron concentration (c(Fe)) of 100 mM and volume of 13.5 μl. The immobilized SF sample was prepared with a mannitol solution (10% w/v). The SF sample was measured in a container with dimension of 3 × 3 × 1.5 mm3 in a cuboid shape. The measurement grid had a size of 25 × 25 × 13 in FOV of size 25 × 25 × 13 mm3 leading to an overscan of the physical FOV of 19.2 × 19.2 × 9.6 mm^3^ ^48^. The SF measurements were acquired with the same aforementioned drive and gradient fields for image acquisition.

### Image reconstruction and analysis by magnetic particle imaging

The images were reconstructed to 25 × 25 × 13 voxels via Kaczmarz’s algorithm with Tikhonov regularization in ParaVision 6 MPI software (Bruker Biospin, Ettlingen, Germany). The hardware background noise limits the used frequency components to a bandwidth to 0.09–125 MHz so the 3rd harmonic is as well filtered out. A number of 487 frequency components were chosen automatically according to the applied SF and the SNR (SNR = 7) threshold determined from the SF. Frequency components with a mixing order above 25 were removed from the further analysis. For reconstruction, a block average of 20 repetitions was applied to the measurement to reduce visible noise in the images. A regularization of λ = 10^−1^^49^ and five iterations were performed for the final reconstructions. These parameters were kept constant in all reconstructions to exclude the influence of variations in these parameters on the intensity values.

For quantification of iron mass in ex vivo MPI images, they were analysed in MATLAB (Mathworks, Natick, MA, USA). First a 50% cut-off threshold of maximum voxel intensity value was applied to the 3D dataset; to eliminate background noise and artefacts, and to minimize the effect of image blurring due to the regularization in image reconstruction^44^. Thereafter the calculation of iron mass in AAA was performed by integration of overall iron in volume of interest (VOI) over the AAA.

### Quantification by magnetic particle spectroscopy

Ex vivo MPS measurements of harvested abdominal aortas (n = 32) were performed using a commercial magnetic particle spectrometer (Bruker, Germany) with a sinusoidal magnetic signal excitation using an amplitude of 25 mT, a frequency of 25 kHz and a sample temperature of 37 °C. The nonlinear magnetization response of MNPs in AAA was measured for 10 s by a pickup coil (sensitivity: 10^–12^ A m^2^). For accurate MPS iron mass quantification, the reference was chosen according to the harmonic ratio A5/A3 of measured sample, immobilized (freeze-dried in 10% mannitol) or in water dispersion and the background signal of the empty sample holder was subtracted from the MPS spectra. For quantitation, the amplitude of the 3rd harmonic of the MPS spectra of measured samples was normalized to the amplitude of the known reference sample.

### Histological analysis of aortic aneurysms and aortic aneurysm morphometry

Histological analysis was performed in the same region of the aorta that was imaged in MRI and MPI. Aortic aneurysm samples were divided in half for paraffin- and cryosectioning, tissues were either processed overnight in MorFFFix (MORPHISTO, Frankfurt am Main, Germany) or frozen at -20° C. 9 µm thick sections of the vessels were stained with Perls` Prussian Blue staining, Miller’s Elastica van Gieson staining, Hematoxylin and Eosin staining. Resulting histological slices were scanned and photographed using a light microscope (Keyence BZ-X800, Keyence Corporation of America, USA). The morphometrical analysis of the aortic region was performed using Keyence BZ-X800 Analyzer software (Keyence BZ-X800, Keyence Corporation of America, USA). To measure the iron oxide percentage in the tissue in a single digitized image, the color profile of iron oxide as seen using Perls’ Prussian Blue was set as reference. All structures within this specific color profile were automatically recorded and divided by the overall tissue area in order to acquire the iron oxide ratio.

### Immunofluorescence analysis

Immunofluorescence staining was performed to assess the localization of macrophages. Frozen AAA samples fixed in optimal cutting temperature compound (OCT) at − 25° were cut into 9 µm thick cryosections and subsequently mounted on SuperFrost microscope slides (Thermo Scientific). The slides were first incubated overnight at 4° using a monoclonal CD68 antibody (Rat anti-Mouse CD68, clone FA-11, Bio-Rad, 1:100) diluted in Dako REAL Antibody Diluent (Dako, Denmark) and subsequently washed with phosphate-buffered saline (PBS, pH 7.4) three times. Slides were incubated with polyclonal secondary antibody AlexaFluor 568 (Goat anti Rat IgG, Thermo Fisher Scientific, Germany, 1:200) for one hour at room temperature, followed by counterstaining and mounting with (DAPI Solution, Roti—Mount FlourCare (CARL ROTH, Germany). Co-localization of macrophages and Perl’s Prussian Blue positive areas were assessed in serials section of AAAs.

### Elemental bioimaging by means of laser ablation-inductively coupled plasma-mass spectrometry for spatial localization of iron

AAA samples were cut at − 25 °C into 9 µm cryosections and immediately mounted on SuperFrost adhesion slides (Thermo Scientific). The LA-ICP-MS analysis was performed with a LSX 213 G2 + laser system (CETAC Technologies, Omaha, USA) equipped with a two volume HelEx II cell connected via Tygon tubing to an ICPMS-2030 (Shimadzu, Kyoto, Japan). Samples were ablated via line-by-line scan with a spot size of 7 µm, a scan speed of 21 µm/s and 800 mL/min He as transport gas. The analysis was performed in collision gas mode with He as collision gas and 50 ms integration time for the ^57^Fe isotope. For the quantification of Fe, matrix-matched standards based on gelatin were used. Nine gelatin standards (10% w/w) including a blank, were spiked with different Fe concentrations ranging from 1 to 5.000 µg/g. Averaged intensities of the scanned lines of the standards showed a good linear correlation with a regression coefficient R^2^ = 0.9999 within this concentration range. Limit of detection (LOD) and limit of quantification (LOQ), calculated with the 3σ- and 10σ-criteria, were 26 µg/g and 86 µg/g Fe. The quantification and visualization were performed with an in-house developed software (WWU Münster, Münster, Germany).

### Statistical analysis

For the comparison of continuous variables, a Student’s t test (unpaired, two-tailed) was applied. p < 0.05 was regarded to be statistically significant.

## Data Availability

The datasets generated during and/or analysed during the current study are available from the corresponding author on reasonable request.
